# The Role of Pitx2 in Maintaining the Phenotype of Myogenic Precursor Cells in the Extraocular Muscles

**DOI:** 10.1371/journal.pone.0058405

**Published:** 2013-03-07

**Authors:** Sadie L. Hebert, Mark L. Daniel, Linda K. McLoon

**Affiliations:** 1 Department of Ophthalmology and Visual Neurosciences, University of Minnesota, Minneapolis, Minnesota, United States of America; 2 Department of Neuroscience, University of Minnesota, Minneapolis, Minnesota, United States of America; University of Pittsburgh, United States of America

## Abstract

Many differences exist between extraocular muscles (EOM) and non-cranial skeletal muscles. One striking difference is the sparing of EOM in various muscular dystrophies compared to non-cranial skeletal muscles. EOM undergo continuous myonuclear remodeling in normal, uninjured adults, and distinct transcription factors are required for the early determination, development, and maintenance of EOM compared to limb skeletal muscle. Pitx2, a bicoid-like homeobox transcription factor, is required for the development of EOM and the maintenance of characteristic properties of the adult EOM phenotype, but is not required for the development of limb muscle. We hypothesize that these unique properties of EOM contribute to the constitutive differences between EOM and non-craniofacial skeletal muscles. Using flow cytometry, CD34^+^/Sca1^−/^CD45^−/^CD31^−^ cells (EECD34 cells) were isolated from extraocular and limb skeletal muscle and *in vitro*, EOM EECD34 cells proliferated faster than limb muscle EECD34 cells. To further define these myogenic precursor cells from EOM and limb skeletal muscle, they were analyzed for their expression of Pitx2. Western blotting and immunohistochemical data demonstrated that EOM express higher levels of Pitx2 than limb muscle, and 80% of the EECD34 cells expressed Pitx2. siRNA knockdown of Pitx2 expression in EECD34 cells *in vitro* decreased proliferation rates and impaired the ability of EECD34 cells to fuse into multinucleated myotubes. High levels of Pitx2 were retained in dystrophic and aging mouse EOM and the EOM EECD34 cells compared to limb muscle. The differential expression of Pitx2 between EOM and limb skeletal muscle along with the functional changes in response to lower levels of Pitx2 expression in the myogenic precursor cells suggest a role for Pitx2 in the maintenance of constitutive differences between EOM and limb skeletal muscle that may contribute to the sparing of EOM in muscular dystrophies.

## Introduction

A myriad of differences exist between extraocular muscles (EOM) and limb skeletal muscles, so much so that the EOM are considered a distinct allotype [1]. One of the more striking differences between EOM and limb skeletal muscles is the preferential involvement or sparing of EOM in a number of skeletal muscle diseases compared to non-cranial skeletal muscles [2]. For example, EOM are spared, both morphologically and functionally, from the progressive degeneration that occurs in limb skeletal muscles in various muscular dystrophies [3], [4]. Potential causes for the sparing of EOM in muscular dystrophies have been investigated, but none have proven mechanistic [5]–[8]. As a result, the sparing of EOM in muscular dystrophies has been attributed to constitutive differences between EOM and other skeletal muscles [8]. The factor(s) controlling these constitutive differences remain unknown. We hypothesize that two unique properties of EOM – their ability to continuously remodel and their unique requirements for development – contribute to the constitutive differences between EOM and non-craniofacial skeletal muscles.

EOM undergo continuous myonuclear remodeling in normal, uninjured adults [9]–[12]. All skeletal muscle is able to regenerate following injury due to the presence of myogenic stem cells, called satellite cells, which reside outside of multinucleated myofibers in a quiescent state. Upon injury these cells become activated and self-renew, proliferate, and differentiate into myofibers [13]. Unlike limb and body skeletal muscles, normal, uninjured adult EOM contain chronically activated satellite cells, which allow EOM to continuously remodel throughout life [9]–[12]. The presence of chronically activated satellite cells in EOM could be due to either a unique or more abundant population of myogenic precursor cells. Our previous work identified a population of myogenic precursor cells, the EECD34 cells (CD34^+^/Sca1^−/^CD45^−/^CD31^−^), that is more abundant in EOM compared to limb skeletal muscle [14]. This population of myogenic precursor cells is maintained at high levels in EOM and is almost absent in limb skeletal muscle of dystrophic mice, suggesting a potential role for this population of myogenic stem cells in the sparing of EOM in muscular dystrophies [14].

Distinct transcription factors are required for the early determination, development, and maintenance of EOM compared to limb skeletal muscle. Paired-like homeodomain transcription factor 2 (Pitx2) is a bicoid-like homeobox transcription factor that is required for the development of EOM [15]. While Pitx2 does play a role during limb skeletal muscle development, limb skeletal muscles develop in its absence and is therefore not required for their development [16], [17]. In addition to its requirement for the initial development of EOM, Pitx2 is also required for the maintenance of characteristic properties of the adult EOM phenotype. Postnatal skeletal muscle-specific knockout of Pitx2 causes a loss of characteristic expression patterns of myosin heavy chain isoforms (MyHC) in the EOM of the transgenic mice, including loss of expression of the EOM-specific (MYH13) and alpha-cardiac (MYH6) MyHC [18], [19]. In addition, these Pitx2 conditional knockout mice lose the multiply innervated muscle fibers normally found in EOM [19], making the EOM more phenotypically like limb skeletal muscle. These differential requirements for Pitx2 during development and in adulthood may contribute to the constitutive differences between EOM and limb skeletal muscles, the sparing of EOM in muscular dystrophies, and the resistance of EOM to injury and denervation.

Here we test the hypothesis that Pitx2 expression is responsible for functional differences between EOM and limb skeletal muscle myogenic precursor cells. EOM and limb skeletal muscle-derived myogenic precursor cells were analyzed for their expression of Pitx2. Pitx2 levels were modified in myogenic precursor cells from EOM and limb skeletal muscle *in vitro* and changes in their proliferative potential and their ability to fuse into multinucleated myotubes were assessed. Levels of Pitx2 expression were examined in dystrophic mouse EOM and limb skeletal muscle and the myogenic precursor cells from these two muscle groups. If high levels of Pitx2 expression are responsible for maintaining the functional differences between EOM and limb skeletal muscle myogenic precursor cells that contribute to the sparing of EOM in muscular dystrophies, Pitx2 levels should remain high in the EOM of the mouse models of muscular dystrophy.

## Materials and Methods

### Ethics Statement

All experiments adhered to NIH guidelines for the use of animals in research and were approved by the Institutional Animal Care and Usage Committee at the University of Minnesota. All tissue acquisition was obtained with written informed consent. These samples were surgical waste obtained from independent surgeons not involved in this study, and their use was approved by the University of Minnesota and University of Texas Southwestern Medical Center Institutional Review Board and adhered to the principles of the Declaration of Helsinki.

### Mice

C57BL/10 mice were purchased from Harlan. The mdx:utrophin^+/−^ mice, originating from Washington University [20] were maintained as a colony at the University of Minnesota through mdx:utrophin^+/−^ breeding pairs. Mice were housed by Research Animal Resources. All mice were sacrificed at 3 months of age by CO_2_ asphyxiation.

### Human Tissue

Control human EOM specimens were obtained as surgical waste after eye removal for causes unrelated to muscle disease. Mean age was 44.5+/−12.3 years. All tissue acquisition was approved by the University of Minnesota and University of Texas Southwestern Medical Center Institutional Review Board and adhered to the principles of the Declaration of Helsinki. The surgical waste EOM were embedded in 5% tragacanth gum, frozen in liquid nitrogen, and stored at −80°C. Muscles were sectioned at 10 µm and stored at −30°C until immunostained.

### Isolation of Mononuclear Cells

Rectus extraocular (EOM) muscles and tibialis anterior (TA) from 10–12 mice (unless otherwise noted) were dissected, pooled, and placed immediately into cold Dulbecco’s minimal essential medium (DMEM). TA was first minced into small pieces, and then EOM and TA were digested in a collagenase/dispase solution (10 mg/ml collagenase B, 2.4 U/ml Dispase II, 2.5 mM CaCl_2_ in phosphate buffered saline (PBS) at 37°C with periodic trituration to dissociate the tissue. The resulting mononuclear cell suspension was filtered through a 70 µm nylon filter, spun into a pellet by centrifugation at 1000 rpm for 5 minutes, and resuspended in sorter buffer (1% fetal bovine serum, 20 mM ethylenediaminetetraacetic acid (EDTA), 25 mM 4-(2-hydroxyethyl)-1-piperazineethanesulfonic acid (HEPES) in PBS).

### Flow Cytometry

Mononuclear cells isolated directly from leg and extraocular muscle tissue were incubated with antibodies to CD34, Sca1, CD45, and CD31 (BD Biosciences) for 30 minutes at 4°C [14]. For cell sorting, the cells were then washed with sorter buffer, spun down, and resuspended in sorter buffer, with 7-aminoactinomycin D (7-AAD) (BD Biosciences) added to allow dead cells to be excluded from analysis. These live CD34^+^/Sca1^−/^CD45^−/^CD31^−^ cells derived immediately from freshly dissected skeletal muscles (referred to as EECD34 cells) were sorted with a BD FACSAria II into cold proliferation media (DMEM, 10% fetal bovine serum, 10% horse serum, 1% penicillin/streptomycin, 0.5% chick embryo extract).

For flow cytometry including a nuclear protein (Pitx2), isolated mononuclear cells from EOM and TA of a single mouse were stained first with a near-IR fixable live/dead marker (Invitrogen) and then stained for cell surface proteins (CD34, Sca1, CD45, and CD31) as above. Cells were washed, fixed in 4% paraformaldehyde for 5 minutes, and permeabilized in PBS containing 0.25% Triton-X 100. Cells were incubated with an antibody to Pitx2 (Capra Science, 1∶500) in permeabilization buffer for 30 minutes, rinsed, and incubated with a goat anti-rabbit-Pacific Blue secondary antibody (Invitrogen, 1∶100) for 30 minutes. Cells were washed with sorter buffer, spun down, resuspended in sorter buffer, and run on a BD FACSCanto. Flow cytometric analysis was performed with FlowJo software.

### Cell Culture

Cells were plated at a density of 5,000 cells/cm^2^ onto Matrigel (BD Biosciences) coated Permanox 8-well chamber slides (Lab-Tek), 24-well, 48-well, or 96-well dishes. Proliferation media was replaced every other day until cells reached the appropriate density.

### Pitx2 Knockdown

Pitx2 expression was knocked down using small interfering RNA (siRNA) at two different time points to assess the effects of the loss of Pitx2 expression on the proliferation and differentiation potential of EECD34 cells derived from EOM and TA. Cells were transfected with 6 pmoles of scrambled control or Pitx2 specific (MSS207650) Stealth RNAi siRNA (Invitrogen) and 0.6 µl of Lipofectamine RNAiMax (Invitrogen) per 200 µl of antibiotic free proliferation media according to the manufacturer’s instructions at ∼40% confluence for proliferation experiments and ∼60% confluence for differentiation experiments. Cells were incubated with siRNA at 37°C, 5% CO_2_ for 48 hours.

### Cell Proliferation Assays

#### Calcein AM

Calcein AM is a cell-permeant dye used to measure cell viability. The non-fluorescent calcein AM is converted to a green fluorescent calcein when taken up into live cells. The fluorescence values obtained correspond to the number of live cells, with a higher fluorescence value indicating a greater number of live cells. On the indicated days, the cells were washed once with PBS then incubated for 30 minutes at 37°C, 5% CO_2_ with 100 µl of 2 µM Calcein AM (Invitrogen) in PBS. Fluorescence values corresponding to the number of viable cells were obtained using a CytoFluor plate reader (PerSeptive Biosystems) with excitation at 490 nm and emission at 520 nm. Values are the average of triplicate wells corrected for background fluorescence defined as the average value of triplicate wells without cells.

#### EdU (5-ethynyl-2′-deoxyuridine) proliferation assay

Proliferating cells were detected with the Click-iT EdU kit (Invitrogen). Proliferation media containing EdU (20 µM) was added to the cells for 1 hour. Cells were fixed and labeled with Oregon Green 488 according to the manufacturer’s instructions. Cells were mounted in Vectashield Mounting Media with DAPI (Vector Laboratories) and examined by fluorescence microscopy at 20X magnification. A minimum of 200 nuclei from at least 5 fields were counted for each independent experiment.

### Differentiation Assay

At 70–80% confluence or 48 hours following siRNA transfection, the proliferation media was replaced with differentiation media (DMEM, 5% horse serum, 1% penicillin/streptomycin). Differentiation media was replaced every other day. After 3 days in differentiation media, the cells were fixed and stained for desmin and counterstained with hematoxylin [21]–[23]. The fusion index was calculated as the number of nuclei present in myotubes divided by the total number of nuclei. Cells were visualized at 20X magnification. A minimum of 200 nuclei from at least 5 fields were counted for each independent experiment.

### Cell Death Assay

Following siRNA transfection (48 hours) under proliferating conditions, media containing unattached cells was placed into a 5 ml FACS tube. Attached cells were rinsed once with PBS followed by trypsinization. Trypsinized cells were rinsed out of wells with sorter buffer and placed in the corresponding 5 ml FACS tube. Cells were spun into a pellet by centrifugation at 1000 rpm for 5 minutes and resuspended in sorter buffer containing 7-AAD to identify dead cells. Cells were run on a BD FACSCanto. The percentage of dead cells was determined by analysis with FlowJo software.

### Real-time Polymerase Chain Reaction (PCR)

Total RNA was isolated from proliferating cells using TRIzol (Invitrogen) according to the manufacturer’s instructions. 500 ng of RNA was reverse transcribed to cDNA using the Superscript III reagents (Invitrogen) according to the manufacturer’s instructions. A 20 µL RT-qPCR reaction containing cDNA, forward and reverse primers (sequences below), and 1 X iQ *SYBR*® Green supermix (Bio-Rad) was run on an Mastercycler® ep realplex (Eppendorf). Relative quantification was determined using the standard curve method. All samples were normalized to GAPDH.

Pitx2 forward 5′- AGCCACTTTCCAGAGAAACCGCTA -3″.

Pitx2 reverse 5′- TTGCGTTCCCGCTTTCTCCATTTG -3′.

GAPDH forward 5′- TCAACTTTCGATGGTAGTCGCCGT -3′.

GAPDH reverse 5′- TCCTTGGATGTGGTAGCCGTTTCT -3′.

myf5 forward 5′- CCCGAAAGAACAGCAGCTTTGACA -3′.

myf5 reverse 5′- CCACAATGCTGGACAAGCAATCCA -3′.

MyoD forward 5′- CAGGCATGCTGTGTAGTGCAACAA -3′.

MyoD reverse 5′- TATTTCCAACACCTGAGCGAGCGA -3′.

### Western Blotting

Whole tissue lysate samples were obtained by homogenizing EOM and TA on ice in radio-immunoprecipitation assay (RIPA) buffer (Sigma) with Complete® Protease Inhibitor Cocktail (Roche). Homogenized samples were centrifuged at 10,000 rpm for 10 minutes, and supernatants were collected. Cytosolic and nuclear fractionated protein samples were obtained using the Compartmental Protein Extraction Kit (Millipore) according to the manufacturer’s instructions. For protein extracts from mononuclear cells, fractionated protein samples were obtained by following the manufacturer’s protocol for cultured cells. Protein concentrations were determined by bicinchoninic acid (BCA) protein assay (Thermo Scientific). 10–35 µg of protein was separated on 10% or 12% mini-PROTEAN TGX gels (Bio-Rad) and transferred to polyvinylidene fluoride (PVDF) or nitrocellulose. Blots were blocked in 5 X bovine serum albumin (BSA) blocker (Thermo Scientific) for 1 hour at room temperature then incubated with one or more of the following primary antibodies: Pitx2 (Capra Science, 1∶2000), Histone H3 (Abcam, 1∶5000), or GAPDH (Biodesign International, 0.1 µg/ml) overnight at 4°C. The next day blots were washed in Tris-buffered saline and Tween 20 (TBST) (1X TBS, 0.1% Tween 20) and incubated in one or more of the following secondary antibodies: goat anti-rabbit IgG-alkaline phosphatase (Applied Biosystems, 1∶10,000), goat anti-rabbit-IR800 (Rockland, 1∶10,000), or goat anti-mouse-IR700 (Rockland, 1∶10,000) for 1 hour at room temperature. For blots incubated with alkaline phosphatase secondary antibodies, proteins were detected with the Western Star reagents (Applied Biosystems) in a G:BOX (Syngene) with GeneSnap software. Densitometry values were obtained with the GeneTools software (Syngene). Blots incubated with IR secondary antibodies were imaged with an Odyssey Infrared Imaging System (LI-COR Biosciences). Densitometry values were obtained using Odyssey instrument software.

### Cytospin

Following sorting into proliferation media, cells were spun into a pellet by centrifugation at 1000 rpm for 5 minutes. Cells were resuspended in proliferation media at a concentration of 30,000cells/200 µl and were spun onto Colorfrost Plus slides (Fisher) at 800 rpm for 3 minutes using a Cytospin 2 (Shandon). Slides were dried overnight at room temperature.

### Immunohistochemistry

Cultured cells were rinsed with PBS, fixed in 4% paraformaldehyde, blocked with 10% horse serum followed by the Avidin/Biotin blocking kit (Vector Laboratories), and incubated overnight with desmin antibody (Dako, 1∶300). Desmin was detected using the Vectastain ABC kit (Vector Laboratories) and diaminobenzidine. Cells were counterstained with hematoxylin.

Cytospin cells were rehydrated in PBS containing 0.1% Tween 20 (PBST), blocked in 20% goat serum/0.2% BSA in antibody buffer (1X PBS, 0.3% Triton-X 100) for 30 minutes at room temperature, and incubated overnight at 4°C with Pitx2 antibody (Capra Science, 1∶1000) in antibody buffer. The following day, cells were washed in PBST, blocked for 5 minutes in 20% goat serum containing 0.2% BSA in antibody buffer, incubated at room temperature for 1 hour with goat anti-rabbit-AlexaFluor 488 antibody (Jackson ImmunoResearch, 1∶1000) in antibody buffer, rinsed in PBST, and mounted with Vectashield with DAPI (Vector Laboratories).

For immunohistochemistry of tissue sections, mouse globes with rectus muscles attached or mouse TA muscles were dissected, embedded in tragacanth gum, and frozen in liquid nitrogen-cooled methylbutane. 10 µm or 12 µm sections were obtained using a cryostat. All immunohistochemistry began with tissue rehydration in PBS, fixation in ice-cold acetone for 10 minutes, and rinsing in PBS.

For double labeling with Pitx2 and dystrophin, laminin, or MyoD, mouse tissue sections were blocked in 20% goat serum/0.2% BSA in antibody buffer for 30 minutes at room temperature, incubated with Pitx2 antibody (Capra Science, 1∶2000) in antibody buffer for 1 hour at room temperature, rinsed, blocked for 10 minutes in 20% goat serum/0.2% BSA in antibody buffer, and incubated with goat anti-rabbit-AlexaFluor 488 antibody (Jackson ImmunoResearch, 1∶2000) or Rhodamine Red-X-goat anti-rabbit antibody (Jackson ImmunoResearch, 1∶1000) in antibody buffer for 1 hour at room temperature. Sections were then incubated in 10% rabbit serum for 1 hour at room temperature followed by an overnight incubation with AffiniPure Fab fragment goat anti-rabbit IgG (Jackson ImmunoResearch, 1∶100) at 4°C. The following day, sections were incubated with dystrophin antibody (Abcam, 1∶1000) or laminin antibody (Sigma, 1∶1000) in antibody buffer for 1 hour at room temperature or MyoD antibody (Santa Cruz, 1∶50) in antibody buffer overnight at 4°C. Next sections were rinsed, blocked as above, incubated with Rhodamine Red-X-goat anti-rabbit antibody (Jackson ImmunoResearch, 1∶1000) or goat anti-rabbit-AlexaFluor 488 antibody (Jackson ImmunoResearch, 1∶2000) in antibody buffer for 1 hour at room temperature, rinsed, and mounted with Vectashield (Vector Laboratories).

For double labeling with Pitx2 and CD34 or Pax7, mouse or human tissue sections were blocked in 20% goat serum/0.2% BSA in antibody buffer for 30 minutes at room temperature, incubated with Pitx2 antibody (Capra Science, 1∶2000) in antibody buffer for 1 hour at room temperature, rinsed, blocked for 10 minutes in 20% goat serum/0.2% BSA in antibody buffer, and incubated with Rhodamine Red-X-goat anti-rabbit antibody (Jackson ImmunoResearch, 1∶2000) in antibody buffer for 1 hour at room temperature. For CD34 labeling, sections were blocked again in 20% goat serum/0.2% BSA in antibody buffer for 30 minutes at room temperature. Sections were then incubated for 1 hour at room temperature with CD34 antibody (eBiosciences, 1∶200), rinsed, blocked for 10 minutes in 20% goat serum/0.2% BSA in antibody buffer, incubated with goat anti-rat-AlexaFluor 488 antibody (Jackson ImmunoResearch, 1∶400) in antibody buffer for 1 hour at room temperature, rinsed, and mounted with Vectashield (Vector Laboratories). For Pax7 labeling, sections were blocked in 20% donkey serum/0.2% BSA in antibody buffer for 30 minutes at room temperature. Sections were then incubated overnight with Pax7 antibody (DSHB, 1∶100) at 4°C. The following day, sections were rinsed, blocked for 10 minutes in 20% donkey serum/0.2% BSA in antibody buffer, and incubated with donkey anti-mouse-AlexaFluor 488 antibody (Jackson ImmunoResearch, 1∶1000) in antibody buffer for 1 hour at room temperature. Finally sections were rinsed and mounted with Vectashield (Vector Laboratories).

### Statistical Analysis

Data reported are means ± SEM. Data were analyzed with a Student’s *t*-test or one-way analysis of variance (ANOVA) followed by a post-hoc Tukey’s multiple comparison using Prism software (Graphpad). Statistical significance was defined as P<0.05.

## Results

### EOM EECD34 Cells Proliferate Faster than Limb Muscle EECD34 Cells

**Figure 1:**
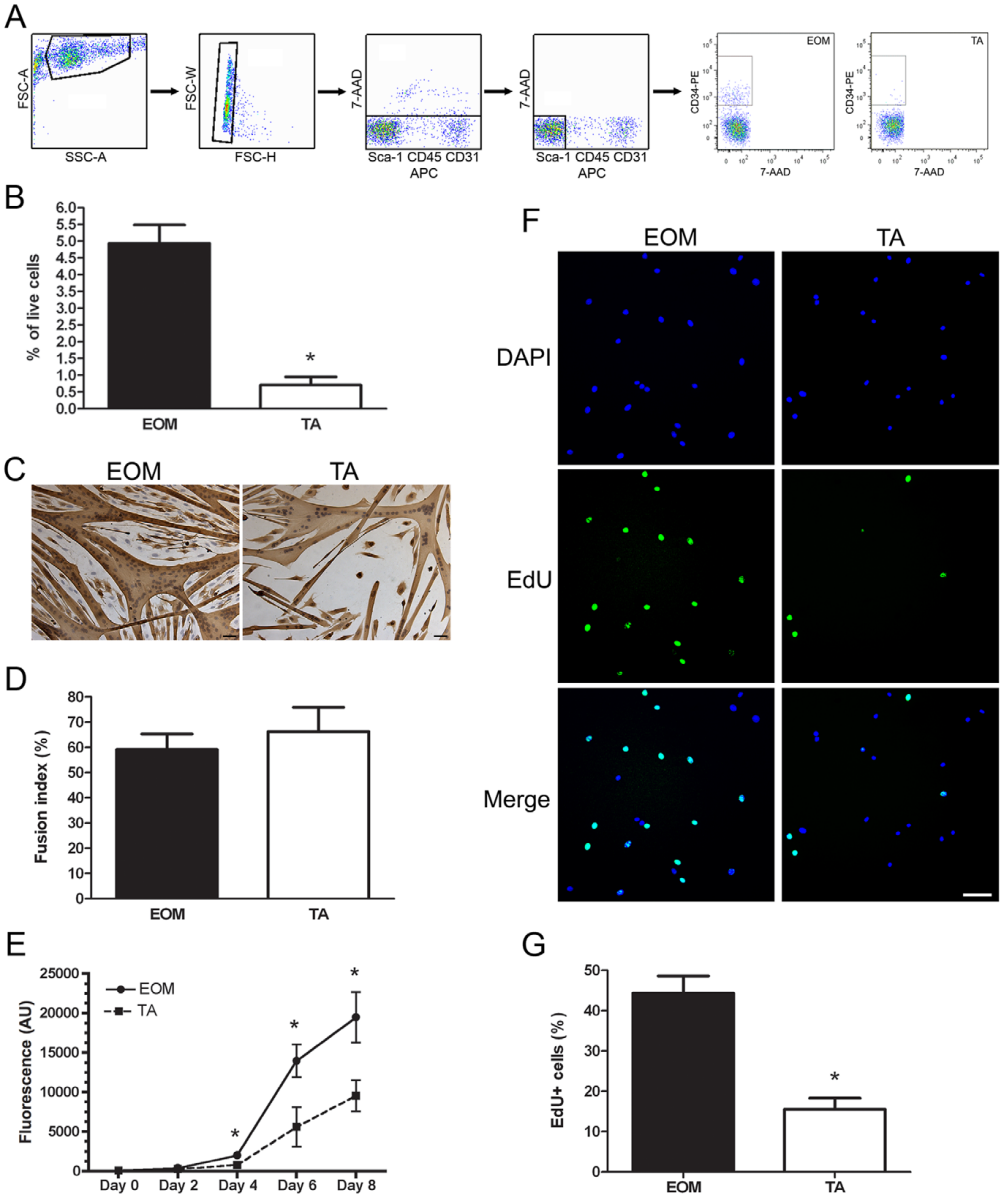
EOM EECD34 cells proliferate faster than limb muscle EECD34 cells. (A) FACS gating strategy for sorting EECD34 cells from wild-type mouse EOM and TA. (B) Percentage of EECD34 cells from wild-type mouse EOM and TA. (C) Fusion of EECD34 cells from wild-type mouse EOM and TA into multinucleated myotubes. (D) Percentage of fused EECD34 cells from wild-type mouse EOM and TA. (E) Time course of wild-type mouse EOM and TA EECD34 cell proliferation. (F) EdU incorporation in EECD34 cells from wild-type mouse EOM and TA. (G) Percentage of wild-type mouse EOM and TA EECD34 cells that have incorporated EdU. Scale bars are 50 µm. * Indicates significant difference from EOM (P≤0.05).

A potential explanation for the sparing of EOM in muscular dystrophy is that there are functional differences in the proliferation and differentiation potential of myogenic precursor cells within EOM and limb skeletal muscle. Using fluorescence activated cell sorting (FACS) CD34^+^/Sca1^−/^CD45^−/^CD31^−^ cells were isolated from EOM and limb skeletal muscle (tibialis anterior, TA). Figure 1A illustrates the FACS gating strategy used to isolate the CD34^+^/Sca1^−/^CD45^−/^CD31^−^ cell population from EOM and TA. The CD34^+^/Sca1^−/^CD45^−/^CD31^−^ cell population is more abundant in EOM than TA, approximately 10-fold more based on the percent of live mononuclear cells, [14] (Figure 1B). This cell population will be referred to as EECD34 cells (Enriched in EOM and CD34^+^).

To determine if there were differences in the ability of EECD34 cells isolated from EOM and TA to fuse into multinucleated myotubes, proliferating EECD34 cells (∼70% confluent) were switched to a reduced serum differentiation media for three days to induce myotube formation. A fusion index was calculated based on the percent of nuclei present in myotubes divided by the total number of nuclei. The majority of EECD34 cells from EOM and TA fused into myotubes with many nuclei (Figure 1C), and the fusion index was similar for EOM and TA EECD34 cells (Figure 1D).

While EECD34 cells from EOM and TA had a similar fusion index, TA EECD34 cells required approximately two additional days in proliferation media to reach ∼70% confluence prompting us to investigate potential differences in the proliferation rate of EECD34 cells isolated from EOM and TA. EECD34 cell proliferation was assessed in two ways. First, EECD34 cell proliferation over time was determined with a calcein AM assay. After four days in proliferation media, there were significantly more EOM EECD34 cells than TA EECD34 cells (Figure 1E). TA EECD34 cell numbers continued to lag behind EOM EECD34 cells for the duration of the time course. It should be noted that the EECD34 cells begin spontaneous fusion into multinucleated myotubes at day 6 (EOM) or day 8 (TA). EOM EECD34 cell density was higher than TA EECD34 density at the time of spontaneous fusion into multinucleated myotubes (data not shown). EECD34 cell proliferation was also assessed with the incorporation of 5-ethynyl-2′-deoxyuridine (EdU), a thymidine analog. At ∼30% confluence, proliferating EECD34 cells were incubated with EdU for one hour followed by immediate detection of EdU-positive cells. Significantly more EdU-positive cells were detected in EOM EECD34 cells compared to the TA EECD34 cells (Figure 1F, G).

### EOM Expresses Higher Levels of Pitx2 than Limb Muscle

**Figure 2:**
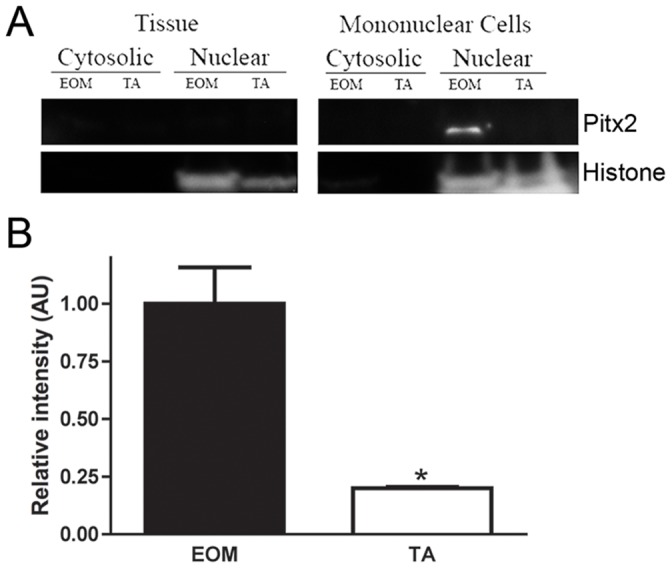
EOM expresses higher levels of Pitx2 than limb muscle. (A) Representative western blot of fractionated whole tissue lysates or isolated mononuclear cell extracts from wild-type mouse EOM and TA probed for Pitx2 and histone. (B) Relative intensity of Pitx2 protein in wild-type mouse EOM and TA normalized to histone. * Indicates significant difference from EOM (P≤0.05).

Based on its critical role in the determination of EOM in early development, Pitx2 protein levels in fractionated (cytosolic and nuclear) tissue lysates and mononuclear cell lysates were assessed by western blotting. Pitx2 protein was not detectable in cytosolic or nuclear fractions of tissue lysates of EOM and TA (Figure 2A). Pitx2 protein levels were significantly higher in nuclear fractions from EOM mononuclear cell lysates compared to nuclear fractions from TA mononuclear cell lysates (Figure 2A, B), with more than a 4-fold difference in expression levels.

**Figure 3:**
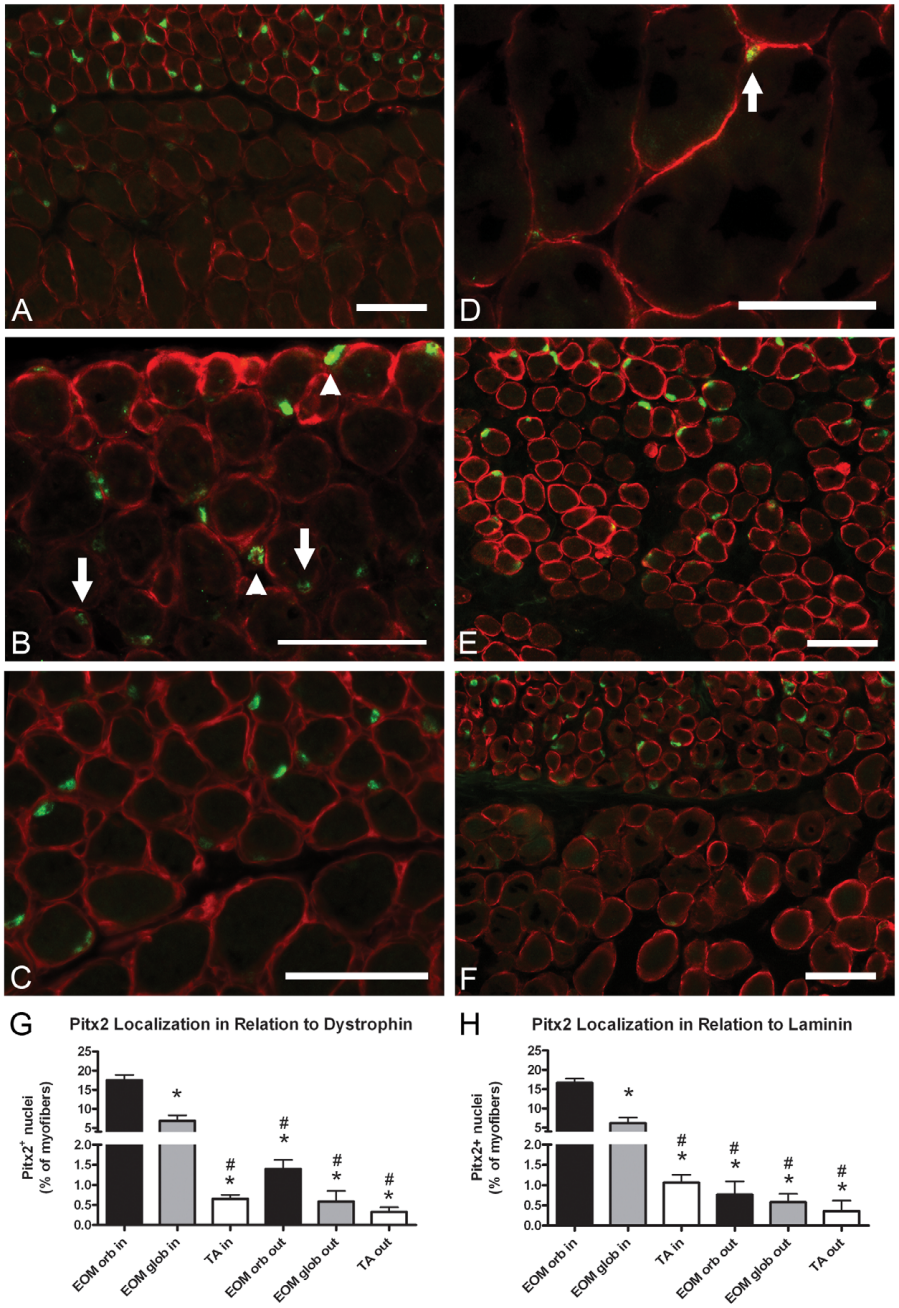
Pitx2-positive nuclei are located within myofibers and outside of myofibers in the satellite cell position. (A, B) Pitx2 (green) and dystrophin (red) localization in wild-type mouse EOM. Arrows point down to Pitx2-positive myonuclei. Arrowheads point up to Pitx2-positive nuclei outside of the dystrophin ring. (C) Pitx2 (green) and laminin (red) localization in wild-type mouse EOM. (D) Pitx2 (green) and dystrophin (red) localization in wild-type mouse TA. Arrow points to a rare Pitx2-positive cell. (E) Pitx2 (green) and dystrophin (red) localization in human EOM. (F) Pitx2 (green) and dystrophin (red) localization in aged (19 months old) wild-type mouse EOM. (G) Percentages of Pitx2-positive nuclei in relation to dystrophin in wild-type mouse EOM and TA. (H) Percentages of Pitx2-positive nuclei in relation to laminin in wild-type mouse EOM and TA. orb, orbital layer of EOM. glob, global layer of EOM. Scale bars are 50 µm. * Indicates significant difference from EOM orbital inside (P≤0.05). # Indicates significant difference from EOM global inside (P≤0.05).

The localization of Pitx2 in tissue sections from EOM and TA was determined by performing immunohistochemistry with double staining for Pitx2 and dystrophin or laminin. Consistent with the western blotting results, Pitx2-positive staining was localized to the nuclei (Figure 3A–F). Pitx2-positive nuclei were significantly more abundant in the orbital layer than the global layer of EOM (Figure 3A, E, F, G, H), with an approximate 3-fold difference in density. Similar patterns of Pitx2 expression were seen in EOM from aged mice (Figure 3F). Most Pitx2-positive nuclei in EOM were found within the dystrophin ring indicating that they are myonuclei (Figure 3B arrows, G). However, approximately 1% percent of the Pitx2-positive nuclei resided outside of the dystrophin ring in EOM (Figure 3B arrowheads, G), and thus are either satellite cells or interstitial cells. EOM immunostained with Pitx2 and laminin (Figure 3C) showed that between 0.5 and 0.75% of Pitx2-positive cells resided outside of the laminin-positive basal lamina (Figure 3H). Thus, Pitx2-positive cells found outside of the dystrophin ring are equally likely to be satellite cells or interstitial cells. Pitx2-positive nuclei in TA were extremely rare (Figure 3D, G, H).

**Figure 4:**
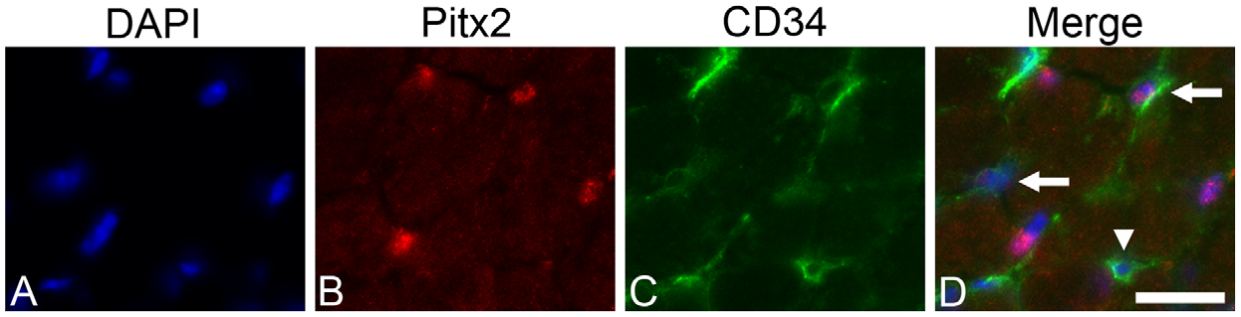
CD34-positive cells express Pitx2 *in vivo*. (A) DAPI, (B) Pitx2, (C) CD34 localizations in mouse EOM. (D) Co-localization of CD34 (green) and Pitx2 (red) in mouse EOM. Scale bar is 20 µm.

The co-expression of CD34 and Pitx2 was confirmed, by finding CD34-positive cells that co-expressed Pitx2 in tissue sections (Figure 4 arrows). As expected by the fact that many other mononuclear cells express CD34 *in vivo*, in addition to the EECD34 cells, CD34-positive cells that did not co-express Pitx2 were also seen (Figure 4 arrowhead).

**Figure 5:**
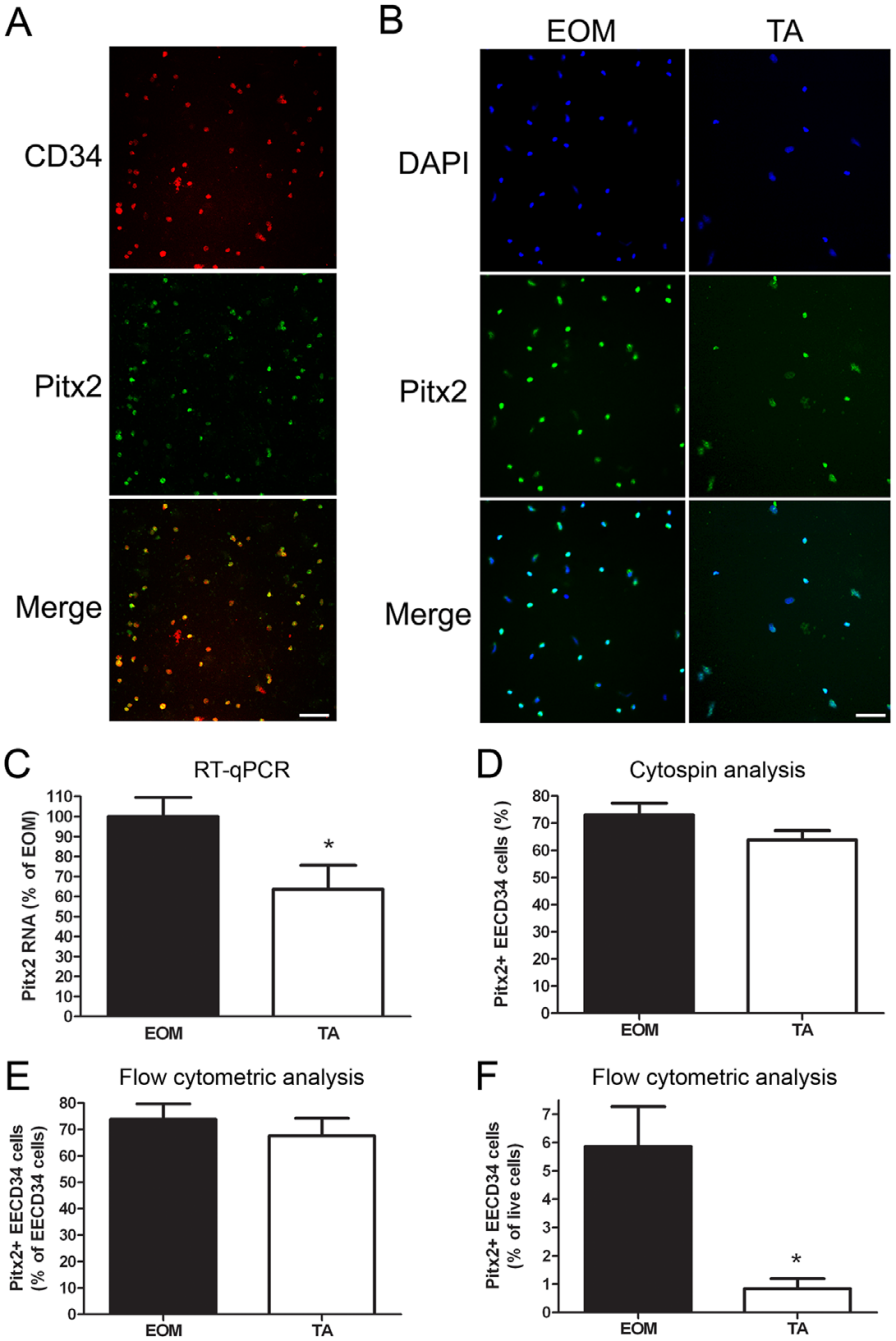
EECD34 cells express Pitx2. (A, B) Pitx2 immunostaining of cytospun EECD34 cells from wild-type mouse EOM and TA. (C) Relative transcript levels of Pitx2 in cultured EECD34 cells from wild-type mouse EOM and TA. (D) Percentage of freshly isolated EECD34 cells from wild-type mouse EOM and TA that are Pitx2-positive determined by immunostaining of cytospun cells. (E) Percentage of freshly isolated EECD34 cells from wild-type mouse EOM and TA that are Pitx2-positive determined by flow cytometry. (F) Percentage of all freshly isolated mononuclear cells from wild-type mouse EOM and TA that are Pitx2-positive EECD34 cells determined by flow cytometry. Scale bars are 50 µm. * Indicates significant difference from EOM (P≤0.05).

When the EECD34 cell populations from EOM and TA were examined, differences in their expression of Pitx2 were seen. The majority of EECD34 cells from both EOM and TA expressed Pitx2 (Figure 5A, B, D, E). Using RT-qPCR, Pitx2 transcript levels in proliferating EOM and TA EECD34 cells were found to be significantly higher in EOM EECD34 cells compared to TA EECD34 cells, with expression levels almost double in the EOM-derived cells (Figure 5C). Immunostaining of freshly isolated EECD34 cells that were cytospun onto glass slides revealed that the percentage of EECD34 cells that were Pitx2-positive was similar between EOM and TA-derived cells (Figure 5B, D). Flow cytometric analysis of freshly isolated cells also showed similar percentages of Pitx2-positive EECD34 cells between EOM and TA (Figure 5E). While a similar percentage of EECD34 cells from EOM and TA are Pitx2-positive, EOM contain 6-fold more Pitx2-positive EECD34 cells than TA (Figure 5F).

### Pitx2-positive Cells do not co-express Pax7

**Figure 6:**
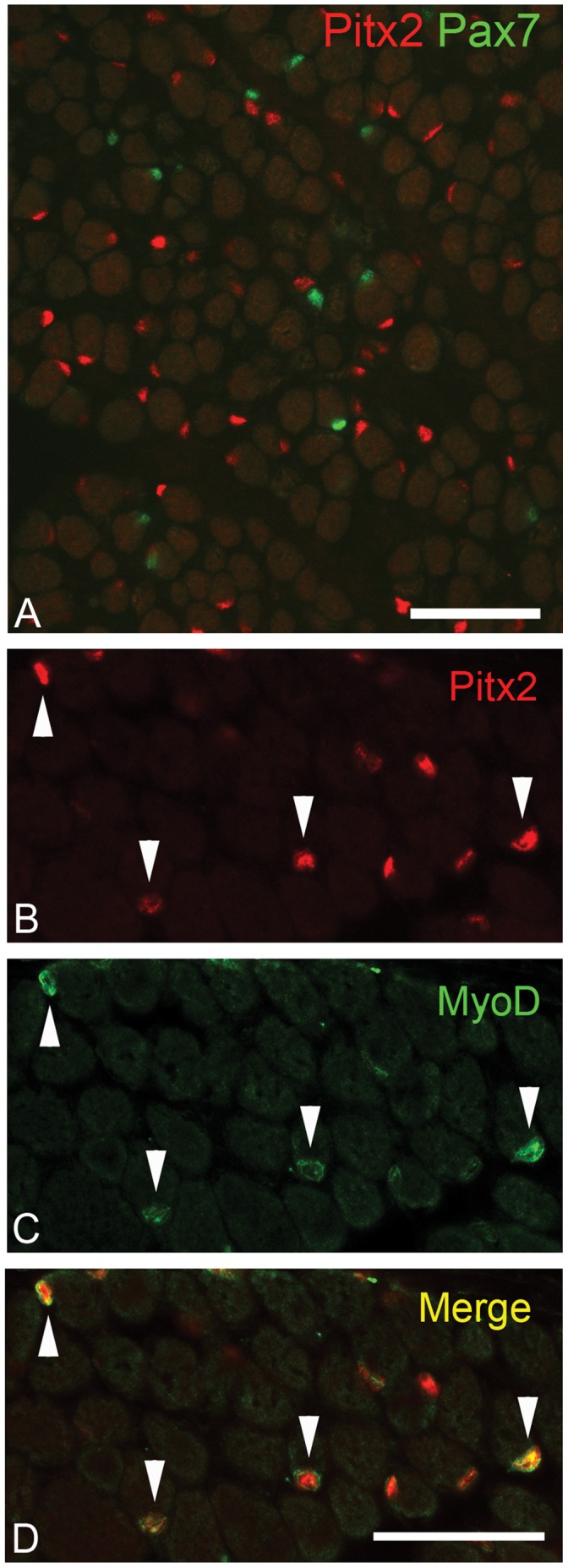
Pitx2-positive cells co-express MyoD, but not Pax7. (A) Pitx2 (red) and Pax7 (green) localization in human EOM. (B) Pitx2 and (C) MyoD localization in wild-type mouse EOM. (D) Co-localization of Pitx2 (red) and MyoD (green) in wild-type mouse EOM. Scale bars are 50 µm.

The potential co-expression of Pitx2 and Pax7 was investigated due to the location of Pitx2-positive cells in the traditional satellite cell position, between the sarcolemma and basal lamina. Double immunostaining of EOM tissue cross-sections revealed that essentially none of the Pitx2-positive cells co-express Pax7 (Figure 6A), with only 2.65% ±0.89 of Pitx2-positive cells co-expressing Pax7. Since Pitx2-positive myonuclei were also seen, the percent of Pax7-positive cells that co-expressed Pitx2 was also assessed and similarly, only a small percentage (14.59% ±6.08) of Pax7-positive cells also expressed Pitx2.

### MyoD Expression in Pitx2-positive Cells

To establish if the Pitx2 cells were in the myogenic lineage *in vivo*, tissue sections were immunostained for the co-expression of MyoD and Pitx2. The majority of cells that expressed MyoD were also Pitx2-positive (Figure 6B–D arrowheads). However, not all Pitx2-positive cells co-expressed MyoD. Thus, it would appear that Pitx2-positive cells are part of the myogenic lineage in EOM, but the expression of Pitx2 does not indicate, a priori, that those cells are differentiating.

### Pitx2 Regulates the Proliferation and Differentiation of EECD34 Cells

**Figure 7:**
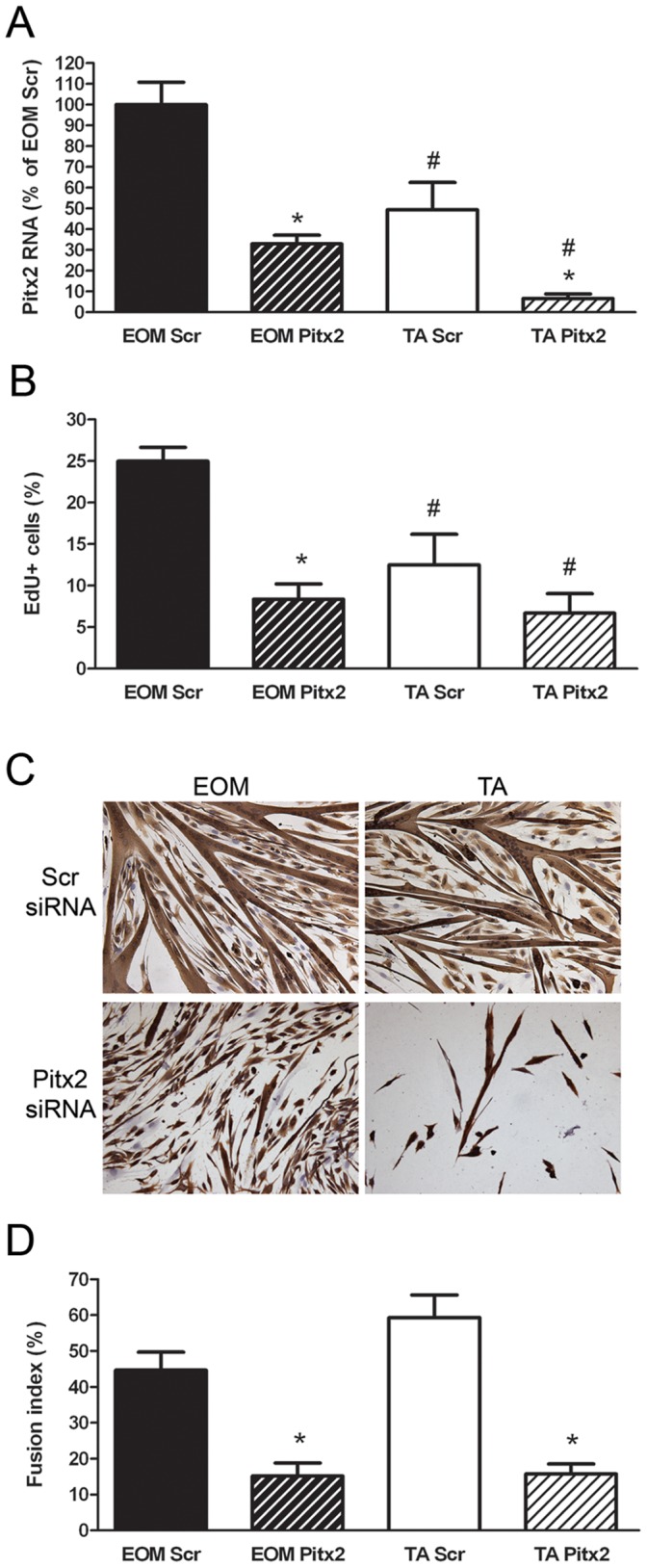
Pitx2 regulates the proliferation and differentiation of EECD34 cells. (A) Relative transcript levels of Pitx2 in scrambled siRNA transfected (Scr) or Pitx2 siRNA transfected (Pitx2) EECD34 cells from wild-type mouse EOM and TA. (B) Percentage of scrambled siRNA transfected (Scr) or Pitx2 siRNA transfected (Pitx2) EECD34 cells from wild-type mouse EOM and TA that have incorporated EdU. (C) Fusion of scrambled siRNA transfected (Scr) or Pitx2 siRNA transfected (Pitx2) EECD34 cells from wild-type mouse EOM and TA into multinucleated myotubes. (D) Percentage of fused scrambled siRNA transfected (Scr) or Pitx2 siRNA transfected (Pitx2) EECD34 cells from wild-type mouse EOM and TA. Scale bars are 50 µm. * Indicates significant difference from EOM (P≤0.05).

To assess the role of Pitx2 in the maintenance of these functional differences between EOM and TA EECD34 cells, the levels of Pitx2 expression in EECD34 cells *in vitro* were knocked down using Pitx2 siRNA. Pitx2 transcript levels were reduced by ∼65% in EOM EECD34 cells and by ∼85% in TA EECD34 cells (Figure 7A). The proliferation and differentiation of EECD34 cells following this reduction in Pitx2 expression was assessed. Reduced Pitx2 expression significantly decreased the proliferation rate of the EOM EECD34 cells and a similar trend was seen for TA EECD34 cells (Figure 7B). Reduction of Pitx2 expression also significantly decreased the ability of both EOM and TA EECD34 cells to fuse and form myotubes (Figure 7C), resulting in a significantly decreased fusion index (Figure 7D). Transcript levels of two potential downstream targets of Pitx2– myf5 and MyoD – were unchanged in EOM and TA EECD34 cells following Pitx2 knockdown (data not shown). Changes in the amount of cell death following Pitx2 knockdown were investigated due to the reduced density of TA EECD34 cells at the end of the fusion assay (Figure 7C), but no differences in the amount of cell death were observed between scrambled and Pitx2 siRNA treated EECD34 cells in EOM or TA (data not shown).

### High Levels of Pitx2 are Retained in Dystrophic Mouse EOM

**Figure 8:**
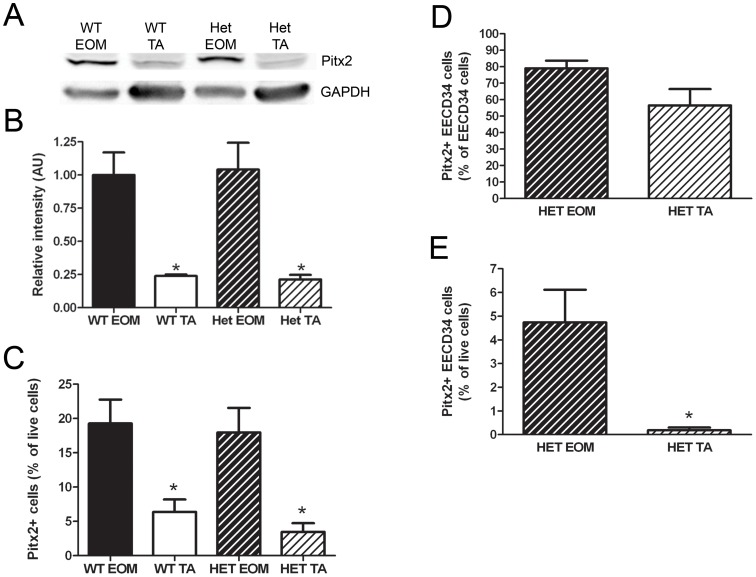
Pitx2 expression is retained in mdx:utrophin^+/−^ EOM. (A) Representative western blot of whole tissue lysates from wild-type and mdx:utrophin^+/−^ (Het) mouse EOM and TA probed for Pitx2 and GAPDH. (B) Relative intensity of Pitx2 protein in wild-type and mdx:utrophin^+/−^ mouse EOM and TA normalized to GAPDH. (C) Percentage of all freshly isolated mononuclear cells from wild-type and mdx:utrophin^+/−^ mouse EOM and TA that are Pitx2-positive. (D) Percentage of freshly isolated EECD34 cells from mdx:utrophin^+/−^ mouse EOM and TA that are Pitx2-positive. (E) Percentage of all freshly isolated mononuclear cells from mdx:utrophin^+/−^ mouse EOM and TA that are Pitx2-positive EECD34 cells. * Indicates significant difference from EOM of the same genotype (P≤0.05).

If high levels of Pitx2 are required for the sparing of EOM in muscular dystrophies, then Pitx2 expression should be retained at high levels in EOM of dystrophic mice. Western blotting was used to determine the levels of Pitx2 in whole tissue lysates from EOM and TA of mdx:utrophin^+/−^ (Het) mice, a mouse model of Duchenne muscular dystrophy, compared to EOM and TA of wild-type mice. Pitx2 protein was expressed at similarly high levels in wild-type and Het mouse EOM and was expressed at 4-fold lower levels in wild-type and Het mouse TA (Figure 8A, B). Flow cytometry was used to analyze the percentage of mononuclear cells that were Pitx2-positive. The percentage of Pitx2-positive cells was similar in wild-type and Het EOM, with approximately 20% of all live cells expressing Pitx2. This was significantly less in wild-type and Het TA, with only approximately 5% of cells expressing Pitx2 (Figure 8C). Analysis of the EECD34 cell population in Het EOM and TA showed similar results to the analysis of the EECD34 cell population in wild-type EOM and TA (compare to Figure 5E, F) with a similar percentage of EECD34 cells expressing Pitx2 in Het EOM and TA (Figure 8D) and significantly more Pitx2-positive EECD34 cells in Het EOM than Het TA (Figure 8E).

## Discussion

EOM are known to be different from non-cranial skeletal muscles in many ways [2], but the factor(s) controlling these differences are not well understood. In this study, we show that the myogenic precursor cells in EOM are functionally different from limb skeletal muscle myogenic precursor cells, adding to the list of differences between EOM and non-cranial skeletal muscle. We previously identified a subpopulation of myogenic precursor cells – the EECD34 cells – that were more abundant in wild-type mouse EOM compared to limb skeletal muscle [14]. As shown in the present study, not only are there differences in the density of these cells, but when placed *in vitro* the EECD34 cells derived from EOM and limb skeletal muscle showed significantly different proliferation rates; the EOM EECD34 cells proliferated at a significantly faster rate than TA EECD34 cells. This is despite having been isolated by flow cytometry using the same criteria, cells with the CD34^+^/Sca1^−/^CD45^−/^CD31^−^ staining profile. Thus, these “same” myogenic precursor cells from EOM and limb skeletal muscle are functionally different. Additionally, when EECD34 cells were maintained in proliferation media and allowed to spontaneously fuse to form multinucleated myotubes, we observed that EOM-derived cells did not begin to fuse until reaching a much higher density than limb skeletal muscle-derived cells, indicating that EOM cells remain in a proliferative state longer than limb skeletal muscle cells. This type of differential proliferation rate has been described for other skeletal muscles. For example, in agreement with our results, a study using all satellite cells derived from either masseter or limb skeletal muscle showed that masseter-derived satellite cells remained in a proliferative state longer than the limb skeletal muscle satellite cells [24]. However, other studies have shown no difference or slower proliferation rates in muscle progenitor cells from masseter compared to limb muscle [25], [26]. In another study, satellite cells isolated from soleus proliferated more rapidly than those from extensor digitorum longus [27], indicating that this type of myogenic precursor cell diversity also occurs among different groups of limb skeletal muscles. Relative to limb skeletal muscle, EOM has also been shown to respond to denervation with increased proliferation of myogenic precursor cells compared to small and/or abortive responses in limb skeletal muscle [28]. Further support for our results is seen in various microarray analyses, which showed that genes related to muscle precursor cell differentiation and the cell cycle were up-regulated in adult EOM compared to limb skeletal muscle [29], [30]. Previous studies discovered that EOM continuously remodel throughout life due to the presence of chronically activated satellite cells [9]–[12]. This process differs in limb skeletal muscle in which satellite cells are only activated in response to injury [13]. This unique ability of EOM to continuously remodel along with the enhanced proliferative capacity of its myogenic precursor cells suggests that the myogenic precursor cells from EOM and limb skeletal muscle are different.

EOM and limb skeletal muscles differ in their embryonic origins and their development requires the expression of distinct genes [15], [31]. A bicoid-like homeobox transcription factor, Pitx2, plays a critical role in the development of a number of tissues and is expressed during development in both cranial and non-cranial skeletal muscle [16], [32], [33]. Interestingly, Pitx2 expression is required for the development of EOM, but while expressed, it is not required for the development of limb skeletal muscle [15], [16], [32]–[34]. Additionally, Pitx2 expression is also required to maintain several of the unique and characteristic properties of mature EOM, including the expression of the EOM-specific and alpha-cardiac myosin heavy chain isoforms as well as multiply-innervated myofibers [18], [19]. This differential requirement for Pitx2 between EOM and limb skeletal muscle suggests that Pitx2 may be one of the factors involved in maintaining functional differences between EOM and limb skeletal muscle myogenic precursor cells.

In adult muscle, we show that Pitx2 protein is expressed at high levels, specifically in the nuclear fraction of cells isolated from EOM compared to limb skeletal muscle. While our work and others showed that transcript levels for Pitx2 are higher in EOM compared to limb skeletal muscles [18], [29], direct comparisons of the levels of Pitx2 protein in EOM and limb skeletal muscle had not been investigated. We examined the localization of Pitx2-positive nuclei in EOM and found the Pitx2-positive nuclei to be preferentially localized to the orbital layer, although the global layer also contained some Pitx2-positive nuclei. In a previous study, Pitx2-positive cells appeared to be equally distributed among the orbital and global layers of EOM [18]; however, the oldest animals used in that study were 6 weeks. Though it should recognize all Pitx2 isoforms, the use of a different Pitx2 antibody in our study may have also contributed to a slightly different staining pattern. While the majority of Pitx2-positive nuclei in EOM were myonuclei, 1% of the Pitx2-positive nuclei were located in satellite cells or interstitial cells. These Pitx2-positive cells outside the myofiber sarcolemma did not express the characteristic satellite cell marker Pax7. This suggests that these Pitx2-positive/Pax7-negative cells represent a distinct population from Pax7-positive satellite cells. Our preliminary data suggests that these may be a more multipotent myogenic precursor cell (Hebert, in preparation). We hypothesize that continued Pitx2 expression in adult EOM may play a role in maintaining activated satellite cells and regulating myofiber remodeling similar to the role it plays in EOM development where Pitx2 is responsible for initiating the myogenic regulatory cascade [15], [16]. This is different from the developmental process in limb skeletal muscle where Pitx2 is not expressed until after the start of the myogenic regulatory cascade and is co-expressed in cells that express Pax3 and Pax7 [17], [35].

One goal of this study was to identify a molecular marker that will more specifically define this population of myogenic precursor cells. Using both flow cytometry and cytospin analyses, we show that the majority of the EECD34 cells are also Pitx2-positive. Their co-expression was confirmed immunohistochemically. Similar to the results comparing percentages of EECD34 cells between EOM and limb skeletal muscles, Pitx2-positive cells are 3 times more abundant in EOM compared to limb skeletal muscle. To examine the potential function of an increased number of Pitx2-positive myogenic precursor cells in adult EOM, we decreased Pitx2 expression *in vitro*, where it significantly altered the function of the EECD34 cells from EOM and limb skeletal muscle. The knockdown of Pitx2 in EOM EECD34 cells slowed their proliferation rate and a similar trend was seen for TA EECD34 cells. A similar effect was seen using myogenic cell lines, where enhanced Pitx2 expression increased proliferation rates and diminished Pitx2 expression reduced proliferation rates [36], [37]. These effects on proliferation rates are likely due to Pitx2 being a downstream target of the Wnt/β-catenin pathway that promotes proliferation during myogenesis through the activation of cell cycle genes [38], [39]. Our studies also showed that the knockdown of Pitx2 reduced the ability of EECD34 cells from both EOM and TA to fuse into multinucleated myotubes. While the reduced density of the TA EECD34 cells following Pitx2 knockdown may have contributed to their reduced ability to fuse into multinucleated myotubes, this was not due to an increase in cell death caused by the loss of Pitx2 expression. To support this view, the density of EOM EECD34 cells was not affected by the knockdown of Pitx2 expression, yet their fusion capacity was also reduced. The progression from proliferating myoblasts to multinucleated myotubes requires the proper expression of the myogenic regulatory factors (i.e. myf5, MyoD, myogenin, MRF4). In EOM, Pitx2 functions upstream of the myogenic regulatory factors, and the expression of these factors during development is sensitive to a loss in Pitx2 expression [15], [34], [40]. These data suggest that a reduction in Pitx2, presumably through regulation of the expression of the myogenic regulatory factors, can influence the fusion capacity of myogenic precursor cells. In our hands, myf5 and MyoD mRNA levels were not different between EOM and TA EECD34 cells and were unchanged by Pitx2 knockdown. In support of these observations, in the various microarray analyses performed comparing EOM and limb skeletal muscle gene signatures, myf5 and MyoD levels were not shown to be different between EOM and limb skeletal muscles [29], [30]. It is interesting to note that many of the MyoD positive cells that are found normally in mature EOM due to their continuous remodeling [10], [12], co-expressed Pitx2. However, not all Pitx2-expressing cells were MyoD-positive. This suggests that a lineage must exist as these precursor cells become committed to differentiation.

It is important to stress that the CD34-positive, Pitx2-positive mononuclear cells do not co-express Pax7, despite their occasional location in the satellite cell position. Pax7 is a well-established marker of quiescent satellite cells [41], but it is also known that there is great heterogeneity in the myogenic precursor cell populations [42], [43]. Ongoing studies are directed at defining other markers that would identify these cells more specifically beyond their co-expression of CD34 and Pitx2. Other quiescent myogenic precursor cell markers have been described. For example, satellite cells positive for CD34 and myf5 were identified previously as early quiescent cells in the myogenic lineage [44]. However, due to the extremely low numbers of Pitx2-positive mononuclear cells in limb muscle, as shown in the present study, and the low levels of myf5 expressed by mature EOM, they most likely represent a different population, with different proliferative and behavioral properties.

Importantly, Pitx2 expression is maintained at high levels in dystrophic mouse EOM and is low in dystrophic limb skeletal muscle, supporting our hypothesis that it may play a role in the sparing of EOM in muscular dystrophies. Similar differences were seen between aging EOM and limb skeletal muscle. The relative numbers of Pitx2-positive cells in dystrophic EOM and limb skeletal muscle mimicked what we saw when we examined the relative numbers of EECD34 cells, which were as abundant in dystrophic mouse EOM as wild-type mouse EOM, but were essentially absent in dystrophic limb skeletal muscle [14]. The maintenance of this specific pool of CD34-positive, Pitx2-positive myogenic precursor cells with increased proliferative potential in adult EOM that is spared in the mdx:utrophin^+/−^ mouse model of muscular dystrophy, and the relative absence of this pool of myogenic precursor cells in affected limb muscles, suggests that these cells may be involved in the ability to maintain muscle normalcy throughout life in these mice and in humans with various forms of muscular dystrophy. Ongoing studies are addressing the signaling pathways that allow these myogenic precursor cells to be preferentially retained in the mature EOM.

In summary, the differential expression of Pitx2 between EOM and limb skeletal muscle along with the functional changes in response to lower levels of Pitx2 expression suggest a role for Pitx2 in the maintenance of constitutive differences between EOM and limb skeletal muscle that may contribute to the sparing of EOM in muscular dystrophies. Pitx2 appears to play a role in maintaining a proliferating pool of myogenic precursor cells, as shown by the gene knockdown studies. This enhanced proliferative capacity may facilitate the repair of damaged EOM tissue in a manner that allows retention of its normal physiological function [3]. Further investigation of the factor(s) that control the enhanced proliferative potential of EOM myogenic precursor cells may aid in understanding the mechanism of EOM sparing in skeletal muscle diseases and provide a target to enhance the regenerative capacity of limb skeletal muscle myogenic precursor cells for the treatment of skeletal muscle diseases.

## References

[1] Lucas CA, Hoh JF (1997) Extraocular Fast Myosin Heavy Chain Expression in the Levator Palpebrae and Retractor Bulbi Muscles. IOVS 38: 2817–2825. 9418735

[2] McLoon LK (2011) The Extraocular Muscles, Chapter 7. In: Kaufman P, Alm A, Levin L, Nilsson S, Ver Hoeve J, et al., editors. Adler’s Physiology of the Eye. Mosby Press. 182–207.

[3] Kaminski HJ, Al-Hakim M, Leigh RJ, Bashar MK, Ruff RL (1992) Extraocular muscles are spared in advanced duchenne dystrophy. Annals of Neurology 32: 586–588. 1456746 10.1002/ana.410320418

[4] Karpati G, Carpenter S, Prescott S (1988) Small-caliber skeletal muscle fibers do not suffer necrosis in mdx mouse dystrophy. Muscle & Nerve 11: 795–803. 3173406 10.1002/mus.880110802

[5] Khurana TS, Prendergast RA, Alameddine HS, Tomé FM, Fardeau M, et al (1995) Absence of Extraocular Muscle Pathology in Duchenne’s Muscular Dystrophy: Role for Calcium Homeostasis in Extraocular Muscle Sparing. J Exp Med 182: 467–475. 7629506 10.1084/jem.182.2.467 PMC2192134

[6] Ragusa RJ, Chow CK, Porter JD (1997) Oxidative stress as a potential pathogenic mechanism in an animal model of Duchenne muscular dystrophy. Neuromuscular Disorders 7: 379–386. 9327402 10.1016/s0960-8966(97)00096-5

[7] Wehling M, Stull JT, McCabe TJ, Tidball JG (1998) Sparing of mdx extraocular muscles from dystrophic pathology is not attributable to normalized concentration or distribution of neuronal nitric oxide synthase. Neuromuscular Disorders 8: 22–29. 9565987 10.1016/s0960-8966(97)00136-3

[8] Porter JD, Merriam AP, Khanna S, Andrade FH, Richmonds CR, et al (2003) Constitutive Properties, Not Molecular Adaptations, Mediate Extraocular Muscle Sparing in Dystrophic Mdx Mice. FASEB J 17: 893–919. 12670877 10.1096/fj.02-0810fje

[9] McLoon LK, Wirtschafter J (2002) Activated satellite cells are present in uninjured extraocular muscles of mature mice. Trans Am Ophthalmol Soc 100: 119–124. 12545684 PMC1358953

[10] McLoon LK, Wirtschafter JD (2002) Continuous myonuclear addition to single extraocular myofibers in uninjured adult rabbits. Muscle & Nerve 25: 348–358. 11870711 10.1002/mus.10056

[11] McLoon LK, Wirtschafter J (2003) Activated Satellite Cells in Extraocular Muscles of Normal Adult Monkeys and Humans. Invest Ophthalmol Vis Sci 44: 1927–1932. 12714625 10.1167/iovs.02-0673 PMC1796845

[12] McLoon LK, Rowe J, Wirtschafter J, McCormick KM (2004) Continuous myofiber remodeling in uninjured extraocular myofibers: myonuclear turnover and evidence for apoptosis. Muscle Nerve 29: 707–715. 15116375 10.1002/mus.20012 PMC1796846

[13] Hawke TJ, Garry DJ (2001) Myogenic Satellite Cells: Physiology to Molecular Biology. J Appl Physiol 91: 534–551. 11457764 10.1152/jappl.2001.91.2.534

[14] Kallestad KM, Hebert SL, McDonald AA, Daniel ML, Cu SR, et al (2011) Sparing of extraocular muscle in aging and muscular dystrophies: A myogenic precursor cell hypothesis. Experimental Cell Research 317: 873–885. 21277300 10.1016/j.yexcr.2011.01.018 PMC3072110

[15] Diehl AG, Zareparsi S, Qian M, Khanna R, Angeles R, et al (2006) Extraocular Muscle Morphogenesis and Gene Expression Are Regulated by Pitx2 Gene Dose. IOVS 47: 1785–1793. 10.1167/iovs.05-1424 16638982

[16] Shih HP, Gross MK, Kioussi C (2007) Cranial muscle defects of Pitx2 mutants result from specification defects in the first branchial arch. Proc Natl Acad Sci U S A 104: 5907–5912. 17384148 10.1073/pnas.0701122104 PMC1851590

[17] Shih HP, Gross MK, Kioussi C (2007) Expression pattern of the homeodomain transcription factor Pitx2 during muscle development. Gene Expression Patterns 7: 441–451. 17166778 10.1016/j.modgep.2006.11.004

[18] Zhou Y, Cheng G, Dieter L, Hjalt TA, Andrade FH, et al (2009) An Altered Phenotype in a Conditional Knockout of Pitx2 in Extraocular Muscle. IOVS 50: 4531–4541. 10.1167/iovs.08-2950 PMC4330467 19407022

[19] Zhou Y, Liu D, Kaminski HJ (2011) Pitx2 Regulates Myosin Heavy Chain Isoform Expression and Multi-Innervation in Extraocular Muscle. J Physiol 589: 4601–4614. 21727215 10.1113/jphysiol.2011.207076 PMC3208227

[20] Grady RM, Teng H, Nichol MC, Cunningham JC, Wilkinson RS, et al (1997) Skeletal and Cardiac Myopathies in Mice Lacking Utrophin and Dystrophin: A Model for Duchenne Muscular Dystrophy. Cell 90: 729–738. 9288752 10.1016/s0092-8674(00)80533-4

[21] Kaufman SJ, Foster RF (1988) Replicating myoblasts express a muscle-specific phenotype. PNAS 85: 9606–9610. 3200845 10.1073/pnas.85.24.9606 PMC282812

[22] Behr T, Fischer P, Müller-Felber W, Schmidt-Achert M, Pongratz D (1994) Myofibrillogenesis in primary tissue cultures of adult human skeletal muscle: expression of desmin, titin, and nebulin. Clin Investig 72: 150–155. 10.1007/BF00184594 8186663

[23] Périé S, Mamchaoui K, Mouly V, Blot S, Bouazza B, et al (2006) Premature proliferative arrest of cricopharyngeal myoblasts in oculo-pharyngeal muscular dystrophy: Therapeutic perspectives of autologous myoblast transplantation. Neuromuscular Disorders 16: 770–781. 17005403 10.1016/j.nmd.2006.07.022

[24] Ono Y, Boldrin L, Knopp P, Morgan JE, Zammit PS (2010) Muscle satellite cells are a functionally heterogeneous population in both somite-derived and branchiomeric muscles. Developmental Biology 337: 29–41. 19835858 10.1016/j.ydbio.2009.10.005 PMC2806517

[25] Pavlath GK, Thaloor D, Rando TA, Cheong M, English AW, et al (1998) Heterogeneity among muscle precursor cells in adult skeletal muscles with differing regenerative capacities. Developmental Dynamics 212: 495–508. 9707323 10.1002/(SICI)1097-0177(199808)212:4<495::AID-AJA3>3.0.CO;2-C

[26] Grefte S, Kuijpers MAR, Kuijpers-Jagtman AM, Torensma R, Von den Hoff JW (2012) Myogenic capacity of muscle progenitor cells from head and limb muscles. European Journal of Oral Sciences 120: 38–45. 22288919 10.1111/j.1600-0722.2011.00920.x

[27] Lagord C, Soulet L, Bonavaud S, Bassaglia Y, Rey C, et al (1998) Differential myogenicity of satellite cells isolated from extensor digitorum longus (EDL) and soleus rat muscles revealed in vitro. Cell and Tissue Research 291: 455–468. 9477302 10.1007/s004410051015

[28] Ugalde I, Christiansen SP, McLoon LK (2005) Botulinum Toxin Treatment of Extraocular Muscles in Rabbits Results in Increased Myofiber Remodeling. IOVS 46: 4114–4120. 10.1167/iovs.05-0549 PMC1847582 16249488

[29] Porter JD, Khanna S, Kaminski HJ, Rao JS, Merriam AP, et al (2001) Extraocular muscle is defined by a fundamentally distinct gene expression profile. PNAS 98: 12062–12067. 11572940 10.1073/pnas.211257298 PMC59827

[30] Fischer MD, Gorospe JR, Felder E, Bogdanovich S, Pedrosa-Domellöf F, et al (2002) Expression profiling reveals metabolic and structural components of extraocular muscles. Physiol Genomics 9: 71–84. 12006673 10.1152/physiolgenomics.00115.2001

[31] Tajbakhsh S, Rocancourt D, Cossu G, Buckingham M (1997) Redefining the Genetic Hierarchies Controlling Skeletal Myogenesis: Pax-3 and Myf-5 Act Upstream of MyoD. Cell 89: 127–138. 9094721 10.1016/s0092-8674(00)80189-0

[32] Gage PJ, Suh H, Camper SA (1999) Dosage requirement of Pitx2 for development of multiple organs. Development 126: 4643–4651. 10498698 10.1242/dev.126.20.4643

[33] Kitamura K, Miura H, Miyagawa-Tomita S, Yanazawa M, Katoh-Fukui Y, et al (1999) Mouse Pitx2 deficiency leads to anomalies of the ventral body wall, heart, extra- and periocular mesoderm and right pulmonary isomerism. Development 126: 5749–5758. 10572050 10.1242/dev.126.24.5749

[34] Zacharias AL, Lewandoski M, Rudnicki MA, Gage PJ (2011) Pitx2 is an upstream activator of extraocular myogenesis and survival. Developmental Biology 349: 395–405. 21035439 10.1016/j.ydbio.2010.10.028 PMC3019256

[35] L’Honoré A, Coulon V, Marcil A, Lebel M, Lafrance-Vanasse J, et al (2007) Sequential expression and redundancy of Pitx2 and Pitx3 genes during muscle development. Developmental Biology 307: 421–433. 17540357 10.1016/j.ydbio.2007.04.034

[36] Martínez-Fernandez S, Hernández-Torres F, Franco D, Lyons GE, Navarro F, et al (2006) Pitx2c overexpression promotes cell proliferation and arrests differentiation in myoblasts. Developmental Dynamics 235: 2930–2939. 16958127 10.1002/dvdy.20924

[37] Gherzi R, Trabucchi M, Ponassi M, Gallouzi I-E, Rosenfeld MG, et al (2010) Akt2-mediated phosphorylation of Pitx2 controls Ccnd1 mRNA decay during muscle cell differentiation. Cell Death & Differentiation 17: 975–983. 20019746 10.1038/cdd.2009.194

[38] Kioussi C, Briata P, Baek SH, Rose DW, Hamblet NS, et al (2002) Identification of a Wnt/Dvl/β-Catenin → Pitx2 Pathway Mediating Cell-Type-Specific Proliferation during Development. Cell 111: 673–685. 12464179 10.1016/s0092-8674(02)01084-x

[39] Baek SH, Kioussi C, Briata P, Wang D, Nguyen HD, et al (2003) Regulated subset of G1 growth-control genes in response to derepression by the Wnt pathway. PNAS 100: 3245–3250. 12629224 10.1073/pnas.0330217100 PMC152277

[40] Sambasivan R, Gayraud-Morel B, Dumas G, Cimper C, Paisant S, et al (2009) Distinct Regulatory Cascades Govern Extraocular and Pharyngeal Arch Muscle Progenitor Cell Fates. Developmental Cell 16: 810–821. 19531352 10.1016/j.devcel.2009.05.008

[41] Seale P, Sabourin LA, Girgis-Gabardo A, Mansouri A, Gruss P, et al (2000) Pax7 Is Required for the Specification of Myogenic Satellite Cells. Cell 102: 777–786. 11030621 10.1016/s0092-8674(00)00066-0

[42] Collins CA, Olsen I, Zammit PS, Heslop L, Petrie A, et al (2005) Stem Cell Function, Self-Renewal, and Behavioral Heterogeneity of Cells from the Adult Muscle Satellite Cell Niche. Cell 122: 289–301. 16051152 10.1016/j.cell.2005.05.010

[43] Biressi S, Rando TA (2010) Heterogeneity in the muscle satellite cell population. Semin Cell Dev Biol 21: 845–854. 20849971 10.1016/j.semcdb.2010.09.003 PMC2967620

[44] Beauchamp JR, Heslop L, Yu DSW, Tajbakhsh S, Kelly RG, et al (2000) Expression of Cd34 and Myf5 Defines the Majority of Quiescent Adult Skeletal Muscle Satellite Cells. J Cell Biol 151: 1221–1234. 11121437 10.1083/jcb.151.6.1221 PMC2190588

